# Nanoparticles exhibit greater accumulation in kidney glomeruli during experimental glomerular kidney disease

**DOI:** 10.14814/phy2.14545

**Published:** 2020-08-12

**Authors:** Gary W. Liu, Jeffrey W. Pippin, Diana G. Eng, Shixian Lv, Stuart J. Shankland, Suzie H. Pun

**Affiliations:** ^1^ Department of Bioengineering and Molecular Engineering & Sciences Institute University of Washington Seattle WA USA; ^2^ Department of Medicine Division of Nephrology University of Washington School of Medicine Seattle WA USA

**Keywords:** biodistribution, glomerular disease, glomeruli, kidney, nanoparticles

## Abstract

Loss and dysfunction of glomerular podocytes result in increased macromolecule permeability through the glomerular filtration barrier and nephrotic syndrome. Current therapies can induce and maintain disease remission, but cause serious and chronic complications. Nanoparticle drug carriers could mitigate these side effects by delivering drugs to the kidneys more efficiently than free drug through tailoring of carrier properties. An important extrinsic factor of nanoparticle biodistribution is local pathophysiology, which may drive greater nanoparticle deposition in certain tissues. Here, we hypothesized that a “leakier” filtration barrier during glomerular kidney disease would increase nanoparticle distribution into the kidneys. We examined the effect of nanoparticle size and disease state on kidney accumulation in male BALB/c mice. The effect of size was tested using a panel of fluorescent polystyrene nanoparticles of size 20–200 nm, due to the relevance of this size range for drug delivery applications.Experimental focal segmental glomerulosclerosis was induced using an anti‐podocyte antibody that causes abrupt podocyte depletion. Nanoparticles were modified with carboxymethyl‐terminated poly(ethylene glycol) for stability and biocompatibility. After intravenous injection, fluorescence from nanoparticles of size 20 and 100 nm, but not 200 nm, was observed in kidney glomeruli and peritubular capillaries. During conditions of experimental focal segmental glomerulosclerosis, the number of fluorescent nanoparticle punctae in kidney glomeruli increased by 1.9‐fold for 20 and 100 nm nanoparticles compared to normal conditions. These findings underscore the importance of understanding and leveraging kidney pathophysiology in engineering new, targeted drug carriers that accumulate more in diseased glomeruli to treat glomerular kidney disease.

## Graphical abstract

During glomerular kidney disease, nanoparticles of size 20 and 100 nm, but not 200 nm, exhibit greater distribution into kidney glomeruli. These physiology‐nanoparticle effects are important to leverage nanotechnology for drug delivery applications to the kidneys.

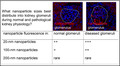

## 1 INTRODUCTION

Chronic kidney disease (CKD) is a major public health problem afflicting nearly 15% of Americans (Saran et al., 2019), often progressing to kidney failure due to a lack of effective interventions. The leading glomerular cause of CKD is focal segmental glomerulosclerosis (FSGS), characterized by loss of kidney podocytes and progressive scarring of kidney glomeruli, the site of kidney filtration (D'Agati, Kaskel, & Falk, 2011; Korbet, 1999). Glucocorticoid steroids have been the frontline FSGS therapy for the past five decades (Schwarz, 2001). However, long‐term glucocorticoid therapy results in serious side effects such as diabetes, cardiovascular disease, and immunosuppression that complicate the disease, and treatment is not effective as 60%–70% of patients are glucocorticoid‐dependent and frequently relapse after steroid cessation (Iijima, Sako, Kamei, & Nozu, 2018; Ruggenenti et al., 2014). These patients require many cycles of high‐dose steroids to achieve remission, which worsen chronic complications relapse after relapse. New treatment strategies with improved safety profiles are urgently needed to induce and maintain disease remission in these patients.

Nanoscale drug delivery systems could address some of these challenges by changing the pharmacokinetic profile of drug cargo through engineering of nanoparticle size, surface charge, composition, and targeting ligands such as antibodies, peptides, and aptamers (Blanco, Shen, & Ferrari, 2015; Rosenblum, Joshi, Tao, Karp, & Peer, 2018). These delivery systems can therefore drive drug delivery to the disease site and mitigate systemic side effects. Indeed, Maeda et al., (2018) have recently shown that nanolipogels functionalized with targeting antibodies enable podocyte‐targeted delivery of drug, mitigating podocyte injury in models of kidney disease. Examination of nanoparticle systems for kidney disease applications is a growing field and could substantially change the standard of treatment. For example, Bruni and colleagues studied poly‐*ε*‐caprolactone and poly(ethylene glycol) (PEG)‐based nanoparticles (NPs) with sizes ranging 5–30 nm for kidney accumulation. While NPs were readily found in urine, suggesting filtration of the materials, they did not appreciably accumulate in the kidneys (Bruni et al., 2017). Williams and colleagues have shown that larger NPs (diameter 350–400 nm) composed of poly(lactic‐*co*‐glycolic acid) and PEG accumulated abundantly in proximal tubule cells, and this behavior was independent of NP surface charge (Williams et al., 2015, 2018). The Davis group has shown that while nanoparticle/nucleic acid complexes (diameter 60–100 nm) of cationic cyclodextrin and siRNA accumulate in the glomerular basement membrane (Zuckerman, Choi, Han, & Davis, 2012), PEGylated gold NPs of size ~75 nm accumulate in kidney mesangium (Choi, Zuckerman, Webster, & Davis, 2011). Collectively these reports show that a combination of nanoparticle physicochemical properties such as charge, size, and material composition affects accumulation in the kidneys and specific cell types within the kidneys. However, an important clinical feature of glomerular disease is enhanced permeation of the glomerular filtration barrier, which may significantly alter the pharmacokinetics of nanoscale drug carriers for kidney disease.

The pathophysiological context during disease is often accompanied by inflammation and injury that leads to disease‐specific cues—increased receptor expression, “leakier” vasculature, and disease‐specific enzymes—that can be exploited to drive NP accumulation and drug release at the disease site. An important example is the enhanced permeation and retention effect of nanoscale drug carriers within tumors described by Matsumura and Maeda (Matsumura & Maeda, 1986). This hypothesis has led to the advancement of NPs engineered with defined size, surface charge, and geometry for enhanced drug delivery to tumors (Blanco et al., 2015; Wang, Stayton, Pun, & Convertine, 2015), leading to clinical approval of the nanoscale formulations Doxil^®^ and Abraxane^®^. Similar to the fenestrated vasculature of tumors, the glomerular filtration barrier also comprises an innermost fenestrated endothelium with 70–100 nm fenestrations, in addition to a middle layer comprising negatively charged glomerular basement membrane, and outermost podocyte foot processes (Du, Yu, & Zheng, 2018). Podocytes are injured in glomerular diseases such as FSGS and diabetic nephropathy (Jefferson & Shankland, 2014; Li et al., 2007), which compromises the integrity of the glomerular filtration barrier and renders the barrier more permeable to macromolecules in the blood, resulting in proteinuria characteristic of these diseases. We have previously shown that glomerular kidney disease increases the accumulation of macromolecular polymers (~25 kDa) in kidney tubules (Liu et al., 2018).

In this work we sought to study the kidney distribution behavior of three NP sizes: 20, 100, and 200 nm, in mice with and without experimental FSGS, a model of glomerular disease that induces podocyte loss, glomerulosclerosis, and increased permeation of macromolecules across the glomerular filtration barrier (Eng et al., 2015; Pippin et al., 2015). While nanoparticles ranging 5–400 nm in size have been studied for kidney distribution, we examined the 20–200 nm size regime because it represents nanoparticle sizes directly relevant for drug delivery applications and is well‐characterized for biodistribution in the context of cancer therapy (Blanco et al., 2015; De Jong & Borm, 2008). Smaller nanoparticles (<20 nm) have been described elsewhere (Choi et al., 2007; Du et al., 2017; Wang et al., 2018) and were not examined because these sizes impose limits on drug loading; larger NPs (>200 nm) were not examined due to rapid complement activation and elimination (Fang et al., 2006; Hoshyar, Gray, Han, & Bao, 2016).

Given the importance of podocyte‐secreted VEGF in maintaining glomerular endothelium homeostasis and the observation that glomerular endothelial fenestrae can increase in diameter from 64 to 195 nm in a mouse model of sepsis (Nagata, 2016; Xu et al., 2014), we hypothesized that kidney distribution of 20‐ and 100‐nm NPs, but not 200‐nm NPs, would be sensitive to experimental FSGS. We show that NPs of size 20 and 100 nm predominantly accumulate in kidney glomeruli, while 200‐nm NPs were rarely observed. Furthermore, FSGS enhanced the glomerular accumulation of the 20‐ and 100‐nm NPs.

## 2 MATERIALS AND METHODS

### 2.1 Materials

*N*‐(3‐dimethylaminopropyl)‐*N*′‐ethylcarbodiimide hydrochloride (EDC), *N*‐hydroxysulfosuccinimide (sulfo‐NHS), and red fluorescent (ex/em 580/605 nm) carboxylated polystyrene nanoparticles of size 20, 100, and 200 nm were purchased from ThermoFisher Scientific. Heterobifunctional poly(ethylene glycol) (PEG, MW = 5,000 Da) with amine and carboxymethyl endgroups (NH_2_‐PEG_5000_‐COOH) was purchased from Laysan Bio.

### 2.2 Nanoparticle functionalization with PEG

Nanoparticle PEGylation was performed as previously reported with modifications (Bugiel et al., 2015; Nance, 2017). All fold‐excess notations are in respect to NP carboxylic acids. NPs (20 mg/ml, 50 μl) were suspended in 50 mM MES buffer pH 6.0, sulfo‐NHS and EDC were added in sequence at an excess of 100‐fold and 200‐fold, respectively, and then the reaction was briefly vortexed. The final volume was 300 μl for 20‐nm NPs and 200 μl for 100‐ and 200‐nm NPs. After 30 min end‐over‐end mixing in the dark, NPs were washed with MES buffer, suspended in NH_2_‐PEG_5000_‐COOH at a 200‐fold excess, and then allowed to react overnight with mixing. NPs were washed with deionized H_2_O, suspended in DPBS to 800 μl (20‐nm NPs) or 200 μl (100‐ and 200‐nm NPs), and sterile‐filtered before experiments.

### 2.3 Nanoparticle characterization

For sizing and ζ‐potential measurements, NPs (2 μl) were diluted in 10 mM NaCl pH 7.2 (700 μl) and analyzed using a Malvern Zetasizer Nano ZS. Three independent readings of 10 runs each were performed. NPs were tested for PEGylation and stability by incubating NPs (1 μl) in 10 mM MgCl_2_ (99 μl) overnight at room temperature as previously described (Curtis, Toghani, Wong, & Nance, 2018). Immediately before analysis, ddH_2_O (900 μl) was added to the NPs, and NP size was analyzed as described above.

To determine NP concentration after PEGylation, NPs were diluted 1:100 in ddH_2_O, and fluorescence was analyzed and compared to a standard curve of known NP concentrations ranging 0–200 μg/mL using a Tecan Infinite 200 PRO plate reader.

### 2.4 In vivo biodistribution

Animals were housed in the animal care facility of the University of Washington under specific pathogen‐free (SPF) conditions with ad libitum access to food and water. Animals were ordered directly from The Jackson Laboratory (strain 000651) and kept in social housing in groups of 2–5 same‐sex animals. Animal protocols were approved (4053‐01) by the University of Washington Institutional Animal Care and Use Committee and experiments were carried out in accordance with the ARRIVE guidelines. NP biodistribution was assessed in 9‐week‐old mice (BALB/c, male) with and without experimental focal segmental glomerulosclerosis (FSGS). To induce FSGS, animals were administered a cytotoxic anti‐podocyte antibody on days −1 and 0 (10 mg antibody/20 g mouse) as we have previously reported (Liu et al., 2018). This antibody induces an abrupt loss of glomerular podocytes that manifests as clinical proteinuria in mice. On day 3, normal and FSGS mice were intravenously administered NPs (50 μg, *n* = 5 mice/NP size), and animals were sacrificed on day 6. Mice were perfused with PBS and major organs (heart, lung, liver, spleen, and kidneys) were collected and analyzed for fluorescence using a Xenogen IVIS (ex/em = 570/620 nm). Regions of interest were drawn around each organ to quantify total radiant efficiency. Spot urines were collected throughout the study, and urinary albumin and creatinine were quantified as previously described (Liu et al., 2018; Marshall, Krofft, Pippin, & Shankland, 2010).

### 2.5 Image collection and analysis

Kidneys were removed, butterflied, and fixed in 4% paraformaldehyde/PBS (Affymetrix) for 45 min at room temperature, and then immersed in 30% sucrose/PBS at 4°C overnight. After, kidneys were patted dry, rinsed briefly, and then embedded in OCT compound (Electron Microscopy Sciences) before freezing in a dry ice/ethanol bath and storage at −80°C. Cryosections (10 μm) were thawed, washed in PBS, stained with 4′,6‐diamidino‐2‐phenylindole (DAPI, 1 μg/ml), and mounted with ProLong™ Gold Antifade Mountant (both from ThermoFisher Scientific) as previously described (Chan et al., 2019; Kaverina et al., 2019; Liu et al., 2018).

For fluorescence microscopy analysis, glomeruli were randomly imaged using a Leica TCS SPE II laser‐scanning confocal microscope (Solms, Germany) equipped with an HCX PL APO 40×/1.30 oil objective, at 1,024 × 1,024 pixel format with 8‐bit intensity resolution. Sets of 22 serial images were collected at 0.3‐μm step size. The following acquisition wavelengths were used: DAPI, excitation 405 nm, emission 380–468 nm; red fluorescent nanoparticles, excitation 561 nm, emission 576–644 nm. At least five images of glomeruli were randomly collected from each mouse.

The number of fluorescent nanoparticle punctae per glomerular surface area was quantified by ImageJ analysis. Individual glomeruli were traced and quantified for area using the “measure” function. Within each glomerular cross‐section, the fluorescent punctae from nanoparticles was detected and counted using the “find maxima” function with a noise tolerance of 25. The number of punctae was divided by the glomerular surface area, and nanoparticle punctae/glomerular surface area were averaged for each mouse.

### 2.6 Data analysis

Data analysis was performed using GraphPad Prism software using a significance level of *α* = 0.05. Single comparisons were tested using a Student's *t*‐test.

## 3 RESULTS

**FIGURE 1:**
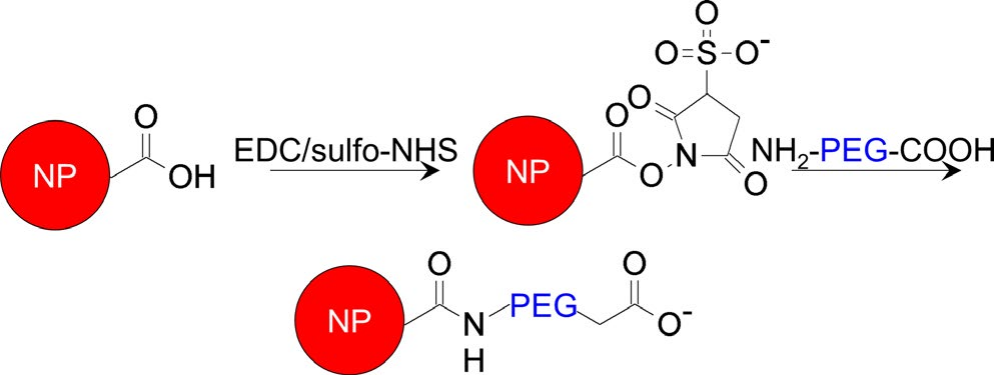
PEG modification of carboxylated nanoparticles. NP surface carboxylic acids are activated via EDC/NHS chemistry and then reacted with the amine group of the bifunctional amine‐ and carboxymethyl‐terminated PEG

We utilized fluorescent, carboxylated polystyrene NPs as model drug carriers for straightforward surface modification, high‐throughput analysis of overall organ distribution by whole‐organ imaging, and spatial interrogation in kidney tissue by confocal microscopy. Serum proteins can absorb to the surface of NPs, increasing their recognition and removal by cells of the reticuloendothelial system by opsonization (Chen et al., 2017; Karmali & Simberg, 2011; Leroux, de Jaeghere, Anner, Doelker, & Gurny, 1995). A widespread strategy to mitigate nonspecific uptake by immune cells is to modify NPs with a dense layer of PEG (Suk, Xu, Kim, Hanes, & Ensign, 2016; Yang et al., 2014). In addition, we and others have shown that greater anionic charge on polymers enhances their accumulation in proximal tubule cells of the kidneys (Borgman et al., 2008; Chen et al., 2019; Kamada et al., 2003; Liu et al., 2018). Therefore, in this study we modified carboxylated fluorescent NPs with anionic, carboxymethyl‐terminated PEG (PEGylation) via EDC/sulfo‐NHS chemistry to improve in vivo pharmacokinetics and leverage anionic charge as a facile targeting method to evaluate kidney accumulation (Figure 1). We did not evaluate cationic materials because they have been described elsewhere (Williams et al., 2015; Zuckerman et al., 2012) and due to concerns of cytotoxicity and inducing nephrotic syndrome (Batsford, Sasaki, Takamiya, & Vogt, 1983; Frohlich, 2012), which may hamper their applicability.

**FIGURE 2:**
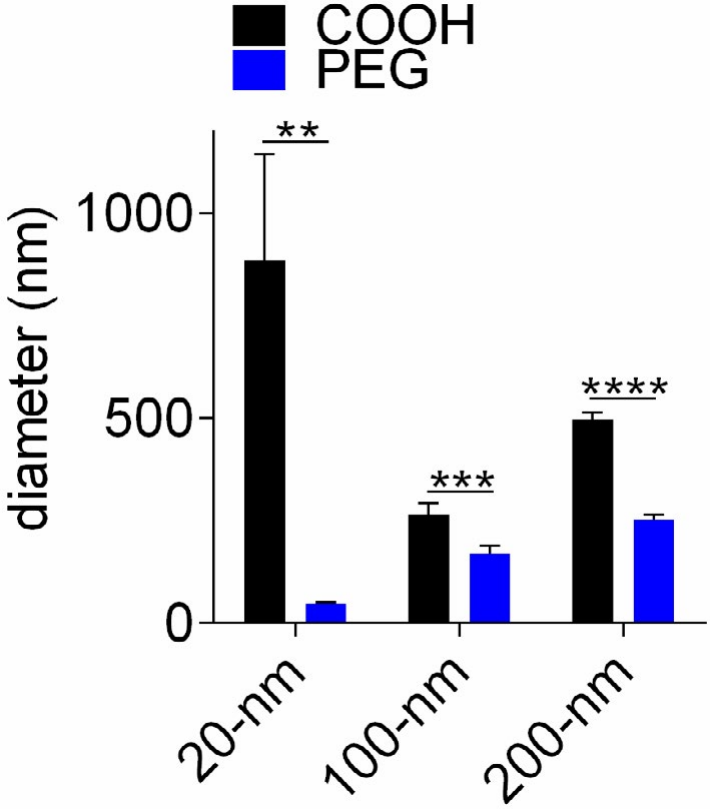
Stability of PEGylated NPs in MgCl_2_. Number average NP diameter of unmodified (black) or PEGylated (blue) NPs challenged with MgCl_2_. Statistical analysis was performed using a two‐tailed Student's *t*‐test. Bars represent means ± *SD*. **p‐value < .01, ***p‐value < .001, ****p‐value < .0001

Because PEGylation modulates NP size and ζ‐potential (the potential at the hydrodynamic shear boundary), the reaction was initially assessed using dynamic light scattering and electrophoretic mobility. PEGylated NPs (‐PEG) exhibited a greater size and more‐neutral ζ‐potential compared to bare (‐COOH) NPs (Table 1). This significant change in ζ‐potential despite reaction with a carboxymethyl‐terminated PEG may be due to the hydrophilic and neutral properties of PEG and its larger chain size (5 kDa) compared to the carboxymethyl group. To further confirm NP PEGylation, NPs were incubated with MgCl_2_, which induces aggregation of unmodified but not PEGylated NPs (Curtis et al., 2018). PEGylated NPs exhibited significantly attenuated aggregation compared to bare NPs (Figure 2, Figure S1). Bare NPs of size 20 nm exhibited the largest aggregates likely due to the greater specific surface area (cm^2^/g) of smaller NPs (Table S1). This observation is consistent with a previous report, which observed an inverse relationship between primary NP size and aggregate size (Chowdhury, Walker, & Mylon, 2013). Collectively, these data indicate successful PEGylation of the NPs.

**TABLE 1:** Nanoparticle diameter and ζ‐potential

Nanoparticle	Diameter (nm)^a^	PDI	ζ‐Potential
20 nm‐COOH	20.6 ± 2.1 nm	0.310	−36.2 ± 2.8 mV
20 nm‐PEG	37.9 ± 3.0 nm	0.395	−19.7 ± 2.3 mV
100 nm‐COOH	86.3 ± 5.2 nm	0.026	−50.9 ± 1.2 mV
100 nm‐PEG	123.2 ± 9.5 nm	0.164	−13.3 ± 0.2 mV
200 nm‐COOH	172.2 ± 1.6 nm	0.032	−64.2 ± 1.6 mV
200 nm‐PEG	236.0 ± 5.7 nm	0.174	−16.1 ± 0.6 mV

Note

Diameter and ζ‐potential are reported as means ± *SD*.

PDI, polydispersity index.

^a^ Number average diameter

**FIGURE 3:**
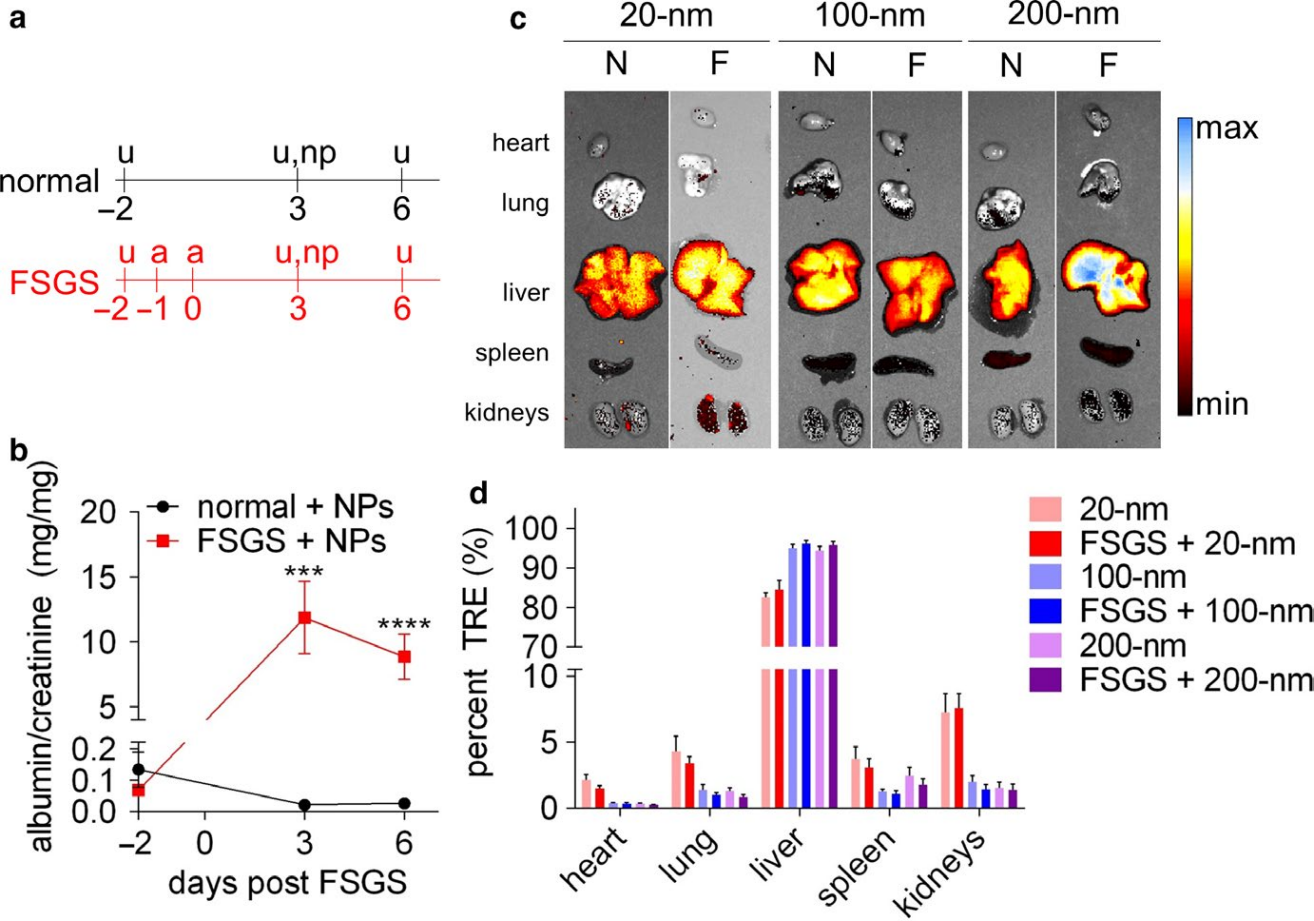
Organ distribution of nanoparticles in normal and experimental FSGS mice. (a) Experimental timeline of treatments. Urine was collected on days −2, 3, and 6 (denoted u), and experimental FSGS was induced in FSGS animals via two sequential injections of cytotoxic anti‐podocyte antibody on days −1 and 0 (denoted a). NPs were injected on day 3 (denoted np). (b) Urinary albumin/creatinine of animals with (red) and without (black) experimental FSGS. Statistical analysis was performed using a two‐tailed Student's *t*‐test. Bars represent means ± *SEM*; *n* = 15 per group. ***p‐value <.001, ****p‐value <.0001. (c) Representative images of major organs 3 days after intravenous administration of fluorescent nanoparticles in normal (N) or experimental FSGS (f) mice. (d). Region of interest quantification of organ fluorescence normalized by total fluorescence. TRE, total radiant efficiency. Bars represent means ± *SD*; *n* = 5 per group

After PEGylation, NPs were injected intravenously into mice (BALB/c, male) with and without experimental focal segmental glomerulosclerosis (FSGS) induced by administration of cytotoxic anti‐podocyte antibodies (Eng et al., 2015; Pippin et al., 2015) and evaluated for whole‐organ biodistribution and kidney distribution. This model of FSGS causes abrupt podocyte loss that manifests as nephrotic‐range albuminuria and glomerular scarring. Because in previous work we showed that kidney accumulation of synthetic polymers is increased in this murine model of FSGS compared to normal mice (Liu et al., 2018), we utilized this model to test the effect of podocytes loss and dysfunction on nanoparticle kidney distribution. FSGS was induced via injection of a cytotoxic anti‐podocyte antibody on days −1 and 0, and all animals were injected with 20‐, 100‐, or 200‐nm PEGylated NPs on day 3 and analyzed for NP biodistribution and kidney distribution on day 6 (Figure 3a). This dosing schedule was selected because animals exhibit peak proteinuria from days 3 to 6 in this model, and NP distribution was evaluated after 3 days because similarly sized gold NPs maintain a relatively consistent kidney distribution for 2–6 days after injection (Li et al., 2018), a timescale relevant for controlled release applications.

While normal mice exhibited urinary albuminuria levels consistent with baseline (day −2), FSGS animals exhibited nephrotic‐range albuminuria (albumin/creatinine ≥ 3.5 g/g) (Nishi et al., 2016) (Figure 3b). Notably, because FSGS animals exhibited nephrotic‐range albuminuria before NP injection that persisted to the study endpoint, NPs pharmacokinetics were subject to a more permeable glomerular filtration barrier throughout the observation period.

To assess NP biodistribution, NP fluorescence in major organs (heart, lung, liver, spleen, kidneys) was analyzed by whole‐organ fluorescence imaging (Figure 3c). Relative tissue distribution was calculated by dividing the individual tissue NP fluorescent signal by the total signal across all analyzed organs of that animal. The relative tissue distribution of 20 nm NPs in the heart, lungs, and kidneys was greater than those of the larger 100 and 200 nm NPs, whereas the larger NPs accumulated more in the liver in both normal and FSGS conditions (Figure 3d) (Li et al., 2018; Lundy, Chen, Toh, & Hsieh, 2016). This observation is consistent with previous reports demonstrating that NP clearance occurs primarily through phagocytosis by Kupffer cells in the liver and that 100 nm particles are more readily internalized by phagocytic cells than smaller particles (Popielarski, Hu‐Lieskovan, French, Triche, & Davis, 2005; Sadauskas et al., 2007; Yu et al., 2012). Relative distribution to the kidneys was also size‐dependent, and was 7.42%, 1.70%, and 1.46%, respectively, for the 20, 100, and 200 nm NPs (averaged across normal and FSGS animals).

**FIGURE 4:**
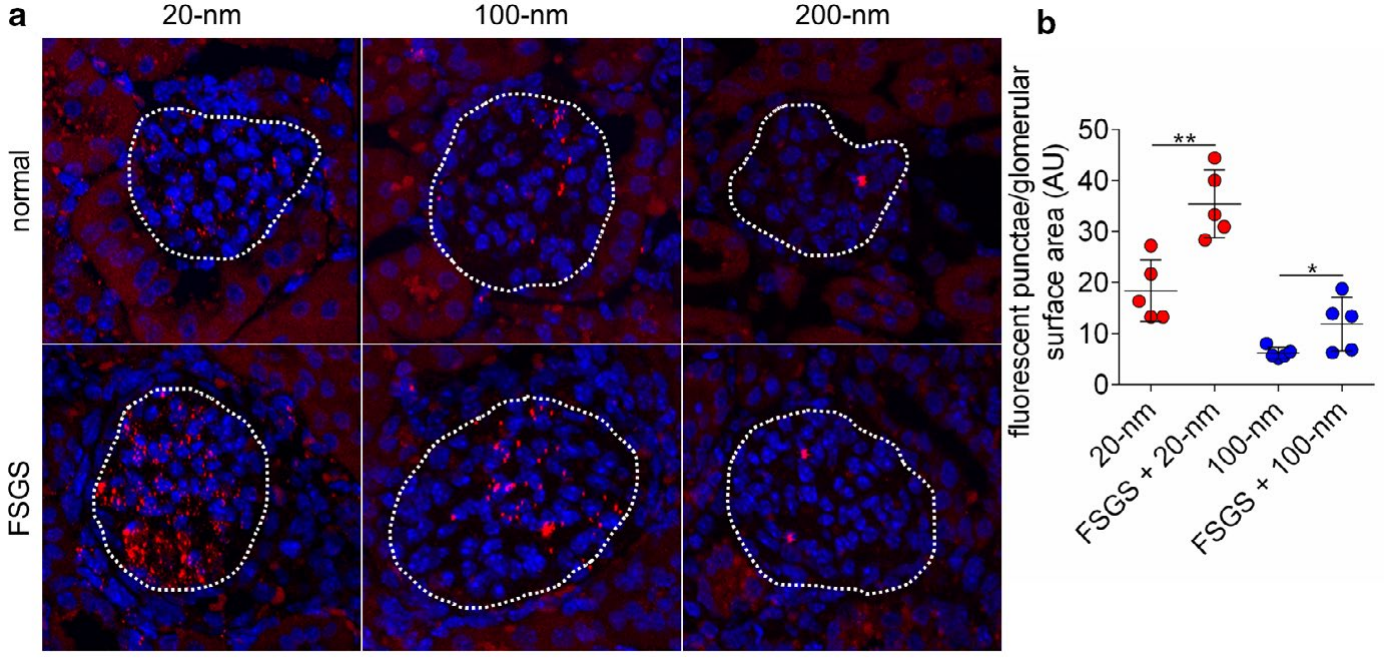
Kidney distribution of nanoparticles in normal and experimental FSGS mice. (a) Representative fluorescent images of kidney glomeruli (denoted by dashed white lines) 3 days after intravenous injection of 20‐, 100‐, or 200‐nm nanoparticles in normal (top row) or experimental FSGS (bottom row) mice. Blue, DAPI; red, nanoparticles. (b) Quantification of the number of nanoparticle fluorescent punctae/glomerular surface area of normal and experimental FSGS mice injected with 20‐ or 100‐nm nanoparticles. AU, arbitrary units. Statistical analysis was performed using a two‐tailed Student's *t*‐test. Bars represent means ± *SD*; *n* = 5 each group. *p‐value < .05, **p‐value < .01

Distribution of the fluorescent NPs in the kidneys was further examined by confocal fluorescence microscopy and found to be both size‐ and disease‐dependent. Nanoparticles of size 20 and 100 nm, but not 200 nm, were consistently found in kidney glomeruli and peritubular capillaries, but not in tubules (Figure S2). Accordingly we focused our analysis of NP accumulation in kidney glomeruli, and quantified nanoparticles by thresholding and quantifying the number of fluorescent punctae using imaging methods. Within glomeruli, NP punctae were located near or within small capillary endothelial cells and podocytes (Figure 4a). Image analysis of individual glomeruli revealed that 20‐nm NPs accumulated more than 100‐nm NPs, and conditions of FSGS augmented glomerular accumulation compared to that of normal animals (Figure 4b; *p*‐value = .0028 and .0475 for 20‐nm and 100‐nm, respectively). Without normalization for glomerular surface area, an average of 30.8 and 62.5 fluorescent punctae of the 20‐nm NPs and an average of 9.3 and 21.5 fluorescent punctae of the 100‐nm NPs were observed in the glomeruli of normal and experimental FSGS mice, respectively. Mice treated with 200 nm NPs were not analyzed, as these NPs were rarely found in the kidneys and glomeruli.

## 4 DISCUSSION

Here we examined the organ and kidney distribution behavior of NPs sized 20, 100, and 200 nm that were functionalized with carboxymethyl‐terminated PEG. While 20‐ and 100‐nm NPs were observed to accumulate within kidney glomeruli and peritubular cells, 200‐nm NPs were rarely observed within the kidneys. Disease conditions of podocyte injury and loss resulted in greater glomerular accumulation of 20‐ and 100‐nm NPs compared to that of normal animals. It is important to note that FSGS is a heterogeneous lesion, where subsections of a subset of kidney glomeruli are injured. In this model, we observed a twofold increase in 20‐ and 100‐nm NP accumulation per glomeruli during FSGS compared to normal conditions. This offers an important clinical advantage as more drug delivery NPs may accumulate in diseased versus healthy glomeruli during FSGS. Future studies could potentially correlate NP accumulation with glomerular injury to further confirm this observation.

Variances due to nanoparticle manufacturing confound direct comparisons of the three nanoparticle sizes with each other: the number of modifiable carboxyls vary (0.14–3.63 COOH/nm^2^) depending on the nanoparticle (Table S1). Previous work examining surface PEG density has revealed that PEG densities as low as 0.1 molecules/nm^2^ extend NP circulation time (Yang et al., 2014), with circulation time increasing with greater PEG density. Interestingly, despite the 20‐nm NPs exhibiting the least number of modifiable carboxyls (and therefore, the lowest PEG density) and the lowest fluorescence intensity (Figure S3), these NPs exhibited the greatest percent fluorescence in the gross kidney and highest number of fluorescent punctae in glomeruli. These observations suggest that NP size likely plays a more predominant role in kidney/glomerular accumulation than PEG density, but a strict comparison between 20‐, 100‐, and 200‐nm NPs is not feasible due to these variations in carboxyl density and fluorescence. However, direct comparisons between disease conditions are still possible within each NP size treatment group, because the same batch of NPs was injected into both normal and FSGS mice.

Because we wanted to preserve spatial information and enable high‐throughput analysis, we utilized fluorescence tracking rather than more quantitative methods such as radiolabeling and elemental analysis. Fluorescence is amenable for gross organ analysis by whole‐organ imaging systems and preserves spatial tissue information for interrogation of NP distribution patterns by confocal microscopy. While this method of biodistribution analysis is considered a semiquantitative method due to limitations in fluorescence penetration, previous studies have shown accurate correlations between fluorescence and elemental analysis methods (Tasciotti et al., 2011). The incongruity between gross kidney and glomerular fluorescence may be explained by sensitivity limitations in whole‐organ imaging systems. We have previously demonstrated that the kidney accumulation differences of anionic polymers, which accumulate in proximal tubule cells, may be robustly quantified by whole‐organ imaging systems. This is likely due to the fact that the great majority of kidney tissue is comprised of proximal or distal convoluted tubule cells (Park et al., 2018), which provide a large and detectable reservoir for the polymers. In contrast, the NPs studied here distribute into glomeruli, which are a minority population in the kidneys and may explain why the more subtle differences in glomerular NP distribution were detectable only by microscopic tissue examination.

While we have previously reported greater proximal tubule accumulation of anionic polymers of molecular weight ~25 kDa during experimental FSGS (Liu et al., 2018), the NPs studied here do not significantly accumulate in tubules. This is especially interesting, as we had modified the NPs with anionic carboxymethyl‐terminated PEG to ostensibly mediate proximal tubule internalization. Instead, NPs were principally found in kidney glomeruli and peritubular capillaries, suggesting that these materials are too large, even during FSGS, to be filtered across the damaged glomerular filtration barrier and accumulate in tubules. This is supported by the observations that 100‐nm NPs accumulate less than 20 nm in kidney glomeruli, yet albumin (~67 kDa) was abundant in the urine. Our findings and others collectively suggest that while NPs may be useful to target the populations of kidney cells proximal to and including the glomerular filtration barrier, for example, podocytes, mesangial cells, and endothelial cells (Choi et al., 2011; Maeda et al., 2018; Zuckerman et al., 2012), polymeric delivery systems may be more effective to target cells that reside beyond the filtration barrier, for example, parietal epithelial cells and proximal tubule cells (Chen et al., 2019; Kamada et al., 2003; Liu et al., 2018).

Within the glomeruli, the 20‐ and 100‐nm NPs were observed to be adjacent to or uptaken by small capillary endothelial cells and podocytes on the basolateral surface. The increased NP accumulation in glomeruli during FSGS may be due to a combination of several factors, including the following: a more permeable glomerular filtration barrier that permits NPs to enter, but not completely cross, the barrier; podocytes are actively endocytotic and upregulate endocytosis during proteinuria (Chung et al., 2015; Eyre et al., 2007; Inoue & Ishibe, 2015; Yoshikawa et al., 1986); and dysfunction of the endothelial glycocalyx during proteinuria may result in greater glomerular endothelial cell uptake of NPs (Cheng, Kumar, Sridhar, Webster, & Ebong, 2016; Fu, Lee, Chuang, Liu, & He, 2015; Salmon & Satchell, 2012). Another factor may be material composition and/or charge—here we utilized anionic, carboxymethyl‐terminated PEG and observed glomerular accumulation in endothelial cells and podocytes, while others utilizing PEG copolymers or neutral PEG observed little or mostly mesangial cell accumulation (Bruni et al., 2017; Choi et al., 2011). Whether NP accumulation is due to charge or dense PEGylation remains to be determined. Other groups have shown that incorporation of targeting ligands using antibodies and peptides can drive nanoparticle accumulation in podocytes (Maeda et al., 2018), tubules (Wang et al., 2018), and activated endothelial cells (Asgeirsdottir et al., 2008). Here we demonstrate that physicochemical properties, in lieu of targeting ligands, can drive NP accumulation in kidney glomeruli, and quantify the effect of local pathophysiology on NP accumulation. As chronic kidney disease affects multiple kidney cell types, multiple nanoparticle platforms are likely critical to arrest disease progression.

“Anchoring” NPs to the glomeruli could act as local drug depots for controlled release of drug to podocytes and endothelial cells—cell populations that are principally affected during such kidney diseases. How might this be utilized in disease? Glucocorticoids are now known to have various direct effects on podocytes; in vitro, glucocorticoids increase nephrin (slit diaphragm protein) expression and maintain cellular viability and actin filament stability during cell injury models (Wada, Pippin, Marshall, Griffin, & Shankland, 2005; Xing et al., 2006; Yamauchi et al., 2006); in vivo, glucocorticoids mitigate podocyte apoptosis, maintain podocyte foot process structure, and reduce proteinuria, a clinical signature of glomerular filtration barrier dysfunction, in animal models of podocyte injury and loss (Mallipattu et al., 2017; Zhang et al., 2013). Therefore, local NP drug depots of glucocorticoids may be an effective strategy to deliver therapeutic concentrations of drug to podocytes while mitigating systemic side effects.

## 5 CONCLUSIONS

In this work we examined the kidney distribution behavior of 20‐, 100‐, and 200‐nm NPs. NPs of size 20 and 100 nm, but not 200 nm, were consistently found in the kidneys in peritubular capillaries and glomeruli, and glomerular disease increased the glomerular accumulation twofold of the two smaller NP sizes. These findings provide important physiological context for new, targeted strategies to treat glomerular kidney disease that leverages the disease state to drive NP accumulation in injured glomeruli.

## CONFLICT OF INTEREST

The authors have no conflicts of interest to declare.

## AUTHOR CONTRIBUTIONS

G.W.L. and S.H.P. conceived and planned the experiments. G.W.L., J.W.P., and D.G.E. carried out the experiments. S.L. provided chemistry expertise on nanoparticle functionalization and purification. G.W.L. and J.W.P. contributed to the interpretation of results. G.W.L. wrote the paper. S.J.S. and S.H.P. supervised the work. All authors reviewed and approved the manuscript.

## Supporting information



Supplementary Material
