# Supplementary material for: Nanoparticles exhibit greater accumulation in kidney glomeruli during experimental glomerular kidney disease
Source: Physiol Rep. 2020 Aug 12;8(15):e14545. doi: 10.14814/phy2.14545 (PMC7422806; doi:10.14814/phy2.14545)
Supplement: Supplementary file 1 — Supplementary Material [file PHY2-8-e14545-s001.docx]

**SUPPLEMENTARY INFORMATION**

**Nanoparticles exhibit greater accumulation in kidney glomeruli during experimental glomerular kidney disease**

Gary W. Liu^1,†^, Jeffrey W. Pippin^2,†^, Diana G. Eng^2^, Shixian Lv^1^, Stuart J. Shankland^2^, and Suzie H. Pun^1,^*

^1^ Department of Bioengineering and Molecular Engineering & Sciences Institute

University of Washington

3720 15^th^ Ave NE

Seattle, WA 98195 (USA)

^2^ Department of Medicine, Division of Nephrology

University of Washington School of Medicine

750 Republican Street, E-179

Seattle, WA 98109 (USA)

^†^ these authors contributed equally to the work

* to whom correspondence should be addressed

email: spun@uw.edu

**
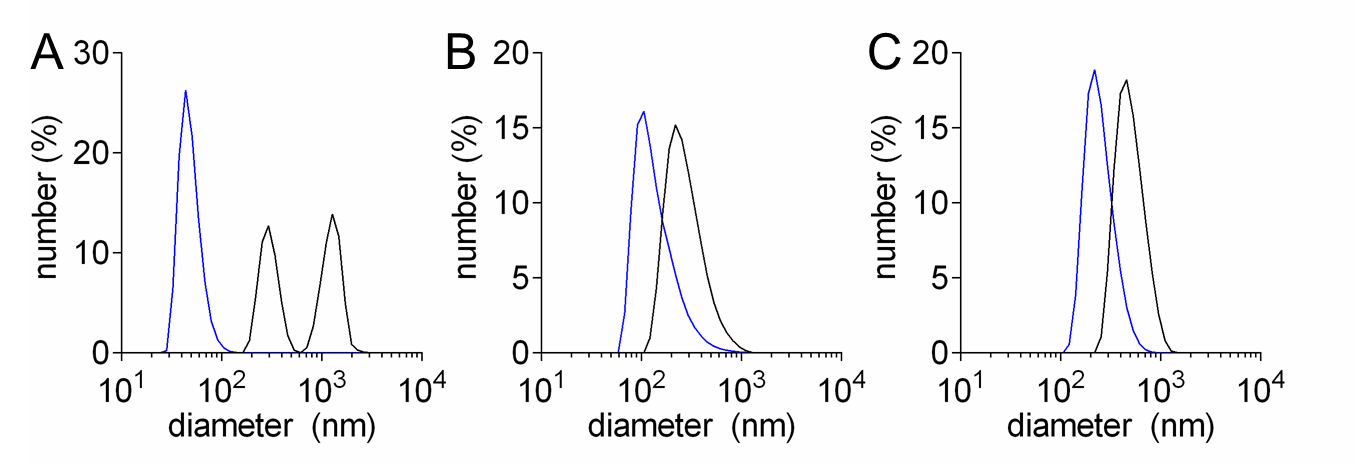
**

**Figure S1. Nanoparticle size distribution.** Representative nanoparticle size distribution by number of PEGylated (blue) and bare (black) nanoparticles after incubation in MgCl_2_. **A.** 20-nm NPs. **B.** 100-nm NPs. **C.** 200-nm NPs.

**
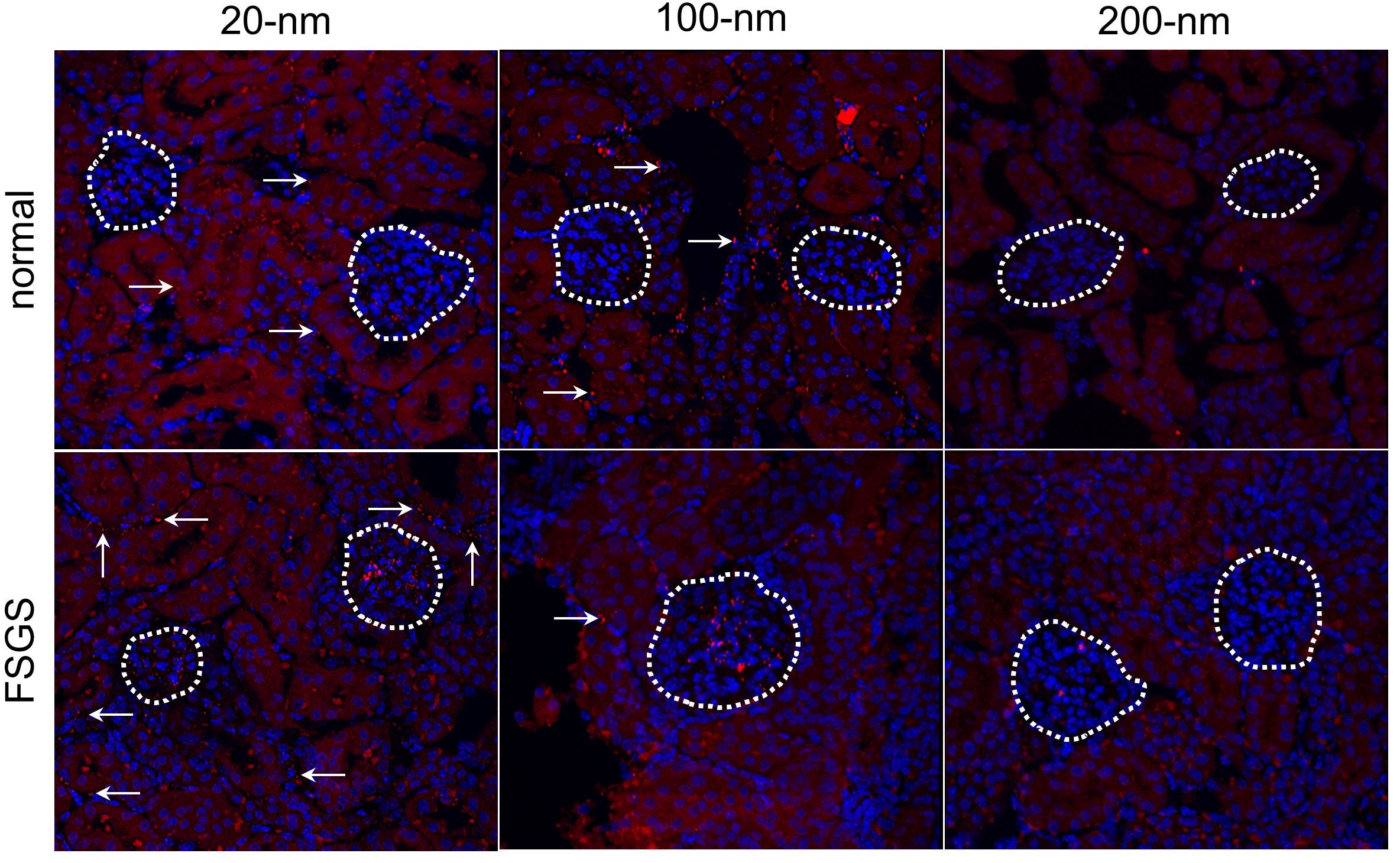
**

**Figure S2. Kidney distribution of nanoparticles in normal and experimental FSGS mice.** Representative fluorescent images of kidney tissue 3 days after intravenous injection of 20-,
100-, or 200-nm nanoparticles in normal (top row) or experimental focal segmental glomerulosclerosis (FSGS, bottom row) mice. Kidney glomeruli are denoted by dashed white lines; peritubular nanoparticle accumulation is denoted by white arrows. Blue, DAPI; red, nanoparticles.





**Figure S3. Nanoparticle fluorescence.** Fluorescence of 20-, 100-, and 200-nm nanoparticles at various concentrations. AU, arbitrary units.

**Table S1.**

|  | **20 nm-COOH** | **100 nm-COOH** | **200 nm-COOH** |
| --- | --- | --- | --- |
| lot | 1922891 | 1985240 | 2041674 |
| charge | 0.0479 mEq/g | 0.3559 mEq/g | 0.0872 mEq/g |
| specific  surface area | 2.0×10^6^ cm^2^/g | 5.9×10^5^ cm^2^/g | 3.0×10^5^ cm^2^/g |
| COOH/nm^2 a^ | 0.14 COOH/nm^2^ | 3.63 COOH/nm^2^ | 1.75 COOH/nm^2^ |
| ^a^ calculated as previously described (Nance, 2017) | | | |

**References**

1. Nance, E. 2017. Brain-Penetrating Nanoparticles for Analysis of the Brain Microenvironment. *Methods Mol Biol,* 1570**,** 91-104.
